# Predictive analysis of multiple future scientific impacts by embedding a heterogeneous network

**DOI:** 10.1371/journal.pone.0274253

**Published:** 2022-09-14

**Authors:** Masanao Ochi, Masanori Shiro, Jun’ichiro Mori, Ichiro Sakata

**Affiliations:** 1 Department of Technology Management for Innovation, The University of Tokyo, Bunkyo, Tokyo, Japan; 2 Human Informatics Interaction Research Institute, National Institute of Advanced Industrial Science and Technology, Tsukuba, Ibaraki, Japan; 3 Mathmatics and Informatics Center, The University of Tokyo, Bunkyo, Tokyo, Japan; University of Sao Paulo, BRAZIL

## Abstract

Identifying promising research as early as possible is vital to determine which research deserves investment. Additionally, developing a technology for automatically predicting future research trends is necessary because of increasing digital publications and research fragmentation. In previous studies, many researchers have performed the prediction of scientific indices using specially designed features for each index. However, this does not capture real research trends. It is necessary to develop a more integrated method to capture actual research trends from various directions. Recent deep learning technology integrates different individual models and makes it easier to construct more general-purpose models. The purpose of this paper is to show the possibility of integrating multiple prediction models for scientific indices by network-based representation learning. This paper will conduct predictive analysis of multiple future scientific impacts by embedding a heterogeneous network and showing that a network embedding method is a promising tool for capturing and expressing scientific trends. Experimental results show that the multiple heterogeneous network embedding improved 1.6 points than a single citation network embedding. Experimental results show better results than baseline for the number of indices, including the author *h*-index, the journal impact factor (JIF), and the Nature Index after three years from publication. These results suggest that distributed representations of a heterogeneous network for scientific papers are the basis for the automatic prediction of scientific trends.

## 1 Introduction

Companies and government agencies must identify promising research and research areas at an early stage to formulate investment strategies for research. Attempts to capture a vast amount of knowledge and to evaluate the direction of future technology development are variously called technology foresight, horizon scanning, technology forecasting, and impact assessment. Recently, government agencies specializing in such activities have been established in many countries. They are trying to use results and knowledge gained to support policymaking. Typical examples include units established within the European Parliament’s Science and Technology Options Assessment (STOA). Methods for investigating the direction of future technological development have so far adopted T-plan methods, Delphi methods, and SWOT analysis based on questionnaires and workshops for experts. However, because of the rapid increase in the number of publications in recent years and the fragmentation of specialized knowledge, analyzing trends of academic research is difficult with only a few members. Furthermore, the demand for more extensive fields and cross-disciplinary research is increasing, making it difficult to predict technological trends that depend on the knowledge of individual researchers. Under these circumstances, attempts have been made actively in recent years to analyze papers and patents directly and to use them for decision making.

Many studies have analyzed the effects of science and technology, mainly expressing the magnitude of the impact as an index. These include, for example, the number of citations in papers, an *h*-index for individual researchers, impact factor for published journals (JIF), and the Nature Index for research organizations (NI). These facilitate a simple comparison of performance differences among multiple subjects merely by showing the performance of papers, researchers, journals, and research organizations at a certain point in time as a simple index. In recent years, there are many criticisms against an excessive emphasis on these indicators. However, such simple indexing is easily understood by non-experts other than researchers, and is widely accepted.

Against this background, predicting these indices has become a significant research problem. There is some research aimed at predicting these indices at an early stage for finding promising research fields in the future. For example, reports have described the prediction of the number of citations [1, 2] and forecast of the *h*-index [3]. These methods are intended to design unique features and models for predicting each index and clarify essential elements and trends in the target index. Specifically, Sasaki *et al*. showed that citing important papers (High PageRank Paper) in an article was correlated in the number of citations in the future. So, is it possible to make a significant impact by merely citing high PageRank papers in science? Of course not. Because which papers an article cites is nothing to do with the actual scientific innovation. In many cases, studies inspired by these high PageRank papers are likely to have a significant science impact, so good papers are expected to cite top PageRank papers. In practice, good research results come from a combination of various entities (inspired research papers, authors, research institutions, specialized fields, journals, etc.) We define a group of such entities that show excellent research results in the future as an emerging research area. The early detection of this emerging research area would be significant evidence for investment decisions by companies and governments.

With the rapid growth of digital publishing, we can use various digital libraries, and some of them make available their Scholarly Big Data (SBD) such as datasets of AMiner, American Physical Society, DBLP, and Microsoft Academic Graph for researchers [4]. Using the information on papers, authors, institutes, venues, fields of study, and other useful entities from SBD, some research constructs knowledge graph for academics (e.g., Microsoft Academic Knowledge Graph [5] and AceKG [6]). Academic knowledge graph enables the development of new systems and approaches in the field of digital libraries to discover hidden relations and semantic-based information such as reading paper or citing paper recommendations.

On the other hand, distributed representation learning methods for the network have been developed recently. Network embedding methods [7–9] that encode each vertex (node) in a network with its vector representation have been studied extensively, e.g., bipartite network [10] and heterogeneous network [11, 12]. Entity and relation network embedding methods [13] which encode each triple (head entity *h* has a relation *r* to tail entity *t*) in a knowledge graph with its vector representation also have been developed. In the context of SBD, some papers apply these embedding methods, e.g., citation recommendation [14, 15] and reading paper recommendation for each user [16], and have succeeded in improving results. One of the relevant research topics in the science of scientific study is to predict the trend of scientific development [17]. So far, many researchers have been working on analyzing and predicting the science trend [18]. To our best knowledge, there are few studies to predict in the future trend applying network embedding method. The network embedding method could integrate various individual scientific indicator prediction models and capture more reasonable scientific trends. So, the purpose of this paper is to show the possibility of integrating multiple prediction models for scientific indices by network-based representation learning.

In this paper, we conduct predictive analysis of multiple future scientific impacts by embedding a heterogeneous network and show the network embedding method is one of the promising tools for capturing and expressing scientific trends. This paper is extended from the conference paper which is “The Representation Extraction for Emerging Research Fields Using an Embedding Method for Heterogeneous Networks [19].” In the conference paper, we presented the basic framework of the method and some initial results on predicting authors’ *h*-indexes. In this paper, we extend the method to predict the *h*-index and the number of citations to paper, the impact factor of a journal, and the Nature Index of a research institution and present the results in a complete form.

### 1.1 Approach

**Fig 1:**
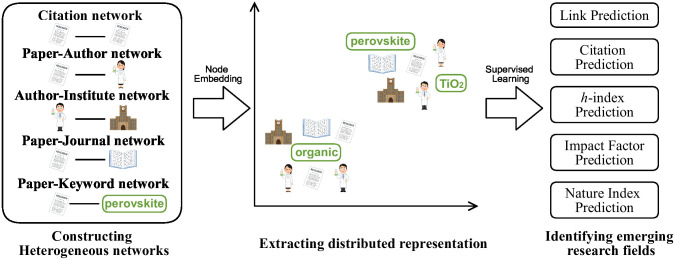
Outline of the method.

The proposed method consists of three parts as shown in Fig 1. First, we construct a heterogeneous network from a scholarly dataset. In this paper, we define the heterogeneous network that is a network with nodes of multiple types. Next, we extract a distributed representation from a heterogeneous network. We identify emerging research areas from the acquired distributed representation, lastly.

We conduct experiments using a scholarly dataset from Scopus. We focus on the research area related to “Solar Cell” from 2006 to 2016. During this period, there were various innovations in solar cells such as inventing perovskite solar cells [20] and the rapid growth of quantum dot solar cells [21] and organic solar cells [22]. This is the reason we select this research area to analyze. We evaluate how our proposed method identify emerging research areas. Our experimental results outperform a baseline method on prediction tasks for identifying emerging research areas. Also, by using a heterogeneous network, our proposed method improve the result better than using a single network. We demonstrate that node embedding methods are effective to find emerging research areas in scholarly data.

### 1.2 Contributions

The contributions of this research are summarized as the following three points.

- We show that the heterogeneous network has complementary effects in a link prediction in scholarly datasets.

We make a heterogeneous network from the scholarly dataset and some of them are sparse, so we confirm the effectiveness such a sparse network on the link prediction task.

- We show that mapping all entities to the same vector space using a node embedding method is effective to predict future research indices.
- We show that our proposed method identify an emerging research area.

Our proposed method embed all entities to the same vector space and predict future research indices. So, we can find the future promising entities as an area.

The rest of this paper is organized as follows. First, Section 1.3 describes previous literature and clarifies the position of this study. In Section 2, we propose a distributed representation extraction method for identifying emerging research areas using heterogeneous network embedding methods. Section 3 describes the data used and the experimental procedure using the proposed method. In the next section, we report details of the experimentally obtained results. Subsequently, we discuss and examine the experimentally obtained results in Section 5. We present conclusions in Section 6.

### 1.3 Previous literature

This study is intended to assess various entities listed in scholarly data in the same vector space and to analyze the relation between extracted entities and multiple future research indices. In this section, we firstly describe the recent rapid growth of digital publishing and various indices for measuring scientific impacts from various aspects. And we explain pros and cons of such simplified indices and it is important to predict them. Next, we explain the method to acquire the distributed representation for the information network and application examples for the SBD. Finally, we clarify points of focus and the significance and novelty of this research, which is the predictive analysis of multiple future scientific impacts by acquiring heterogeneous network distributed representations in the scholarly dataset.

Recently, we can use various digital libraries and some of them make available their SBD such as datasets of AMiner, American Physical Society, DBLP and Microsoft Academic Graph for researchers [4] with the rapid growth of digital publishing. Some research constructs knowledge graph for academic (e.g., Microsoft Academic Knowledge Graph [5] and AceKG [6]) using various information of research papers such as authors, institutes, venues, fields of study and other useful entities from SBD. Academic knowledge graph enables development of new systems and approaches in the field of digital libraries to discover hidden relations and semantic based information such as reading paper or citing paper recommendation.

Thus, research indicators are promising because they have the potential to evaluate the impact of science and technology objectively. On the other hand, several reports point to the abuse of indicators [23, 24]. In particular, universities and government agencies make hiring decisions or give grants based solely on the researchers’ *h*-index or journal impact factor. However, journal impact factors have different citation numbers among research fields, and it is wrong to evaluate all research by this indicator. Therefore, normalized impact factors [25] and other indicators that remove bias have been proposed [26]. There is also an active movement to introduce social evaluation, including the Altmetrics [27]. In this paper, we predict only four representative indicators, however we would not ignore these trends in research indicators development.

Research investigating the impact of science and technology has specifically examined the development of indicators and future predictions. The development of indicators is mainly aimed at quantifying the influence of individual objects: if the subject is a paper, then the number of citations might be used; the author is evaluated by the *h*-index [28]; journals are evaluated by the journal impact factor (JIF) [29]; research institutes are assessed according to the Nature index (NI). Of course, various indicators other than these have been developed, but most of them were for papers and authors.

Research to predict these indices has also been reported. Many studies have already been conducted to predict future *h*-index values [30–33]. Acuna et al. calculated an equation for predicting *h*-index. They showed that five main parameters are fundamentally important for prediction [33]: the number of publications, the current *h*-index value, the number of years since the first publication, the number of types of journals published to date, and the number of papers in top journals. This result demonstrates that the *h*-index is linked with various entities that compose scholarly data, such as papers and submitted journals. Therefore, analyzing scholarly data including multiple entities is important. We address that very task as described in this paper.

Some studies have been undertaken to predict the number of future citations of papers [1, 2, 34, 35]. Among them, Stegehuis et al. and Cao et al. predict the number of citations in the far future considering the number of citations during 1–3 years after publication. In contrast, Sasaki et al. predict the number of citations after three years from publication directly. The task evaluated in this study also predicts the number of citations three years after publication, just as Sasaki et al. did.

Second, since 2014, many researchers have attempted to map networks directly into vector space [7–9]. This trial is still being actively researched and developed in various ways. A typical attempt to deepen is called Graph Convolutional Neural Network (GCN) [36]. However, at present, many reports have described that the scale of the network is about 100,000 nodes. The hierarchy of the neural network is about two layers. Therefore, many difficulties remain in relation to enlargement and deepening. In this paper, we propose a random walk-based node representation extraction method that can be readily scaled up and extended to a heterogeneous network that are constructed based on a scalarly data. A random walk-based node representation extraction method, including nodes of multiple types, has already been studied. The BINE is a technique for obtaining a distributed node representation of a bipartite network [10]. First, for a network with nodes of two types, *U* and *V*, we create a network with only a set of *U* and a network with only a set of *V* considering the secondary proximity between the nodes. It is a technique to execute a random walk on each network and to obtain a distributed representation of nodes. However, because this method maps each node set *U* and *V* onto different vector spaces. Analyzing the proximity of nodes between *U* and *V* in the space is difficult.

Examples of approaches for distributed representation extraction for a heterogeneous network with two or more types of nodes are PTE [12] and metapath2vec [11]. The first, PTE, is a method to acquire three distributed representations on the same vector space for a heterogeneous network connected by three bipartite networks. By contrast, metapath2vec defines the type of random walk across heterogeneous nodes as meta-path(P). It obtains a distributed representation by repeatedly sampling vertices according to every kind of node according to this P. Because metapath2vec performs a random walk along the defined meta-path P, the proximity between specific nodes can be analyzed more appropriately. Actually PTE presents the benefit that each bipartite network is independent, such that another network with some nodes in stock can be added or deleted. This study specifically considers the benefits of PTE and extends it for a scholarly data. Moreover, it examines a proposed method for mapping all nodes onto a vector space from a heterogeneous network defined by multiple bipartite networks. We then evaluate whether the obtained distributed representation is useful for identifying an emerging research area.

On the other hand, some works apply the network embedding methods to SBD. Entity and relation network embedding methods [13] which encode each triple (head entity *h* has a relation *r* to tail entity *t*) in a knowledge graph with its own vector representation also have been developed. In the context of SBD, some papers apply these embedding methods, e.g., citation recommendation [14, 15] and reading paper recommendation for each user [16], and have succeeded in improving result.

One of the important research topics in the science of science study is to predict the trend of scientific development [17]. So far many researchers have been working on analyzing and predicting the science trend [18]. To our best knowledge, there are few studies to analyze the future trend applying network embedding method to the SBD.

## 2 Method

This section describes a proposed method for distributed representation extraction to identify emerging research areas using paper data. The proposed method consists of three parts. One part creates a heterogeneous network from a scholarly dataset, a part that extracts distributed representations from a heterogeneous network, and a part that identifies an emerging research area using the extracted distributed representations. Therefore, we first clarify the role played by each part by explaining the outline of the entire method. Subsequently, we explain details of each part according to the procedure.

### 2.1 Overview of the proposal method

We outline the proposed method in Fig 1. As the figure shows, the proposed method consists of three parts. It consists of creating a heterogeneous network from a scholarly dataset, extracting a distributed representation from a heterogeneous network, and finally executing a task to identify emerging research areas from the acquired distributed representation. Here, we first explain how to create a heterogeneous network from a scholarly dataset; then we describe how to map each node of the created a heterogeneous network to a single vector representation space. Finally, we describe how to apply the core distributed representations to various tasks to identify emerging research areas.

### 2.2 Creation of a heterogeneous network using a scholarly datasets

**Fig 2:**
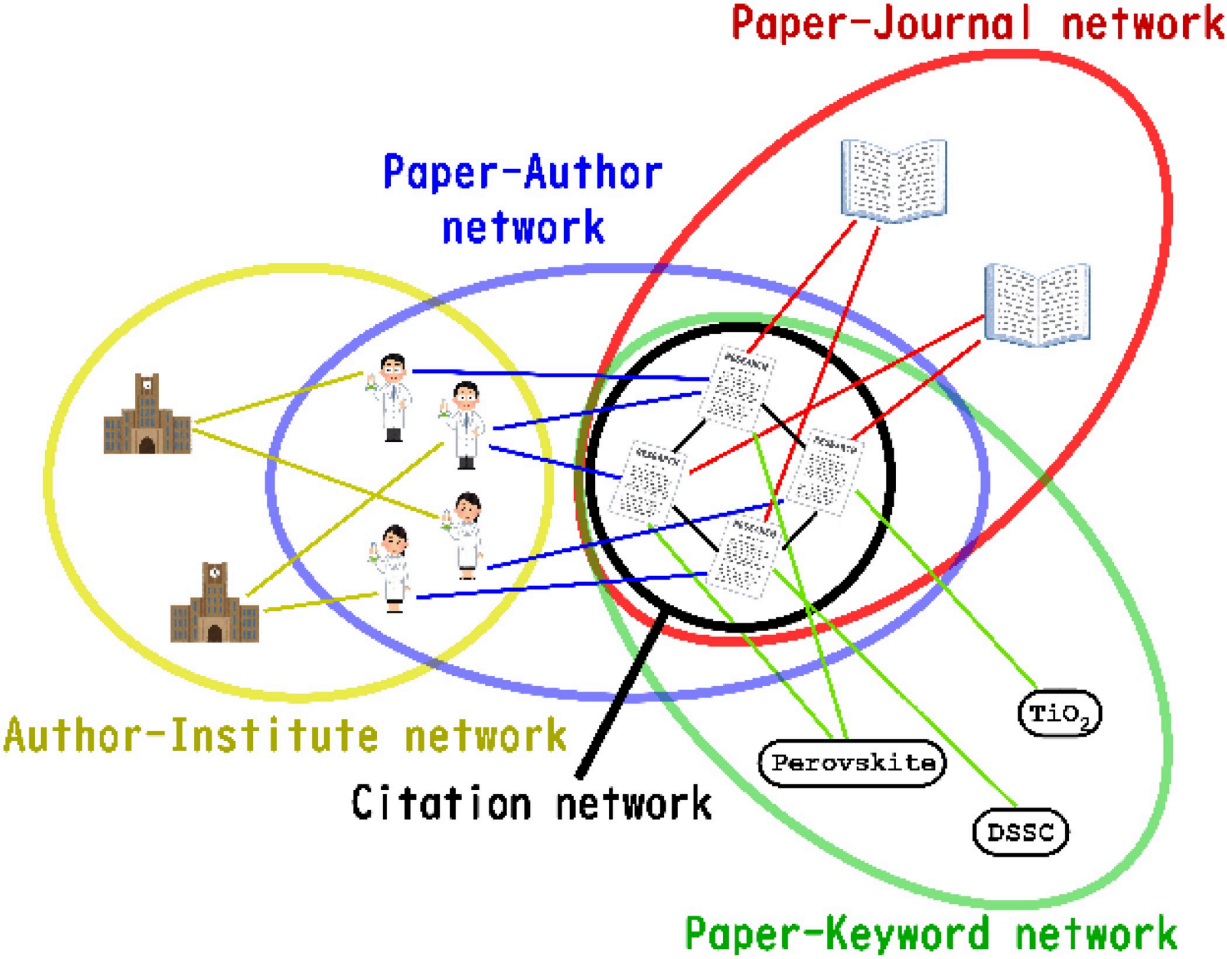
Outline of a heterogeneous network to be created.

Here we describe how to create a heterogeneous network using a scholarly dataset. Fig 2 outlines the heterogeneous network we create. Our method extracts five types of entities which are papers from reference list in the paper, keywords from author provided keyword list, the published journal, authors who write the paper, and institutions where each author belong to. We make edges between each entities as a heterogeneous network and calculate for all papers this extraction method. Each network shares some nodes with other networks. No network shares them all. These various entities described in the paper are reconfigured as a network around the paper. First, the “Citation network” is a network of citation relations among papers. Next, the “Paper–Author network” connects the paper and the author with an edge. However, no edge exists between co-authors. Some connection must be made between the paper and the author. The “Author–Institute network” is an edge connection between the author and the institute to which the author is affiliated. Furthermore, we call the Paper–Keyword network a keyword that is connected with the paper. Finally, we add a “Paper–Journal network” that links the paper to the journal which published it.

Many of these networks are bipartite networks composed of nodes of different types, not homogeneous networks consisting of only a single type of node, such as only papers or authors.

### 2.3 Node embedding method on a heterogeneous network

We describe how to map nodes on a heterogeneous network into the same vector space. First, we represent a set of multiple bipartite networks ***G*** as shown below, where *G* represents a network, *V* denotes a node set, and *E* stands for an edge set.

$$\begin{array}{c} G≔\{G^{l}=\left(V^{l},E^{l}\right)\left|1\leq l\leq |G\right|\} \end{array}$$ (1)

Each *G*^*l*^ is a bipartite network such as the “Author–Institute network” or “Citation network” as shown in Fig 2. Also, |***G***| is the number of bipartite networks. Each network is an undirected network *G*^*l*^ ≔ (*V*^*l*^, *E*^*l*^) composed of nodes and edges. Although no shared edge exists in each network, some nodes are shared with other networks. We consider a method of mapping a certain node *v*_*i*_ ∈ ***V*** on a heterogeneous network set ***G*** to a particular vector $\vec{u_{i}}\in U$ in a vector space. Here, ***V*** represents all nodes *V*^*l*^ on each bipartite network *G*^*l*^.

First, we calculate the edge weight *w*_*ij*_ = *P*(*v*_*j*_|*v*_*i*_) to the node *v*_*j*_ adjacent to the node *v*_*i*_ on a particular network. In this paper, all bipartite networks are undirected graphs, and all edge weights have the same value. We calculate this value by the mapped distributed representation and the following formula. For simplicity, we omitted subscripts *l* for specific networks.

$$\begin{array}{c} \hat{P}\left(v_{j}|v_{i}\right)=\frac{\text{exp}({\vec{u_{j}^{\prime }}}^{T}\cdot \vec{u_{i}})}{\sum_{k\in |V|}\text{exp}\left({\vec{u_{k}^{\prime }}}^{T}\cdot \vec{u_{i}}\right)} \end{array}$$ (2)

Therein, |*V*| is the number of nodes on the network, $\vec{u_{i}}$ is the node mapping vector, and $\vec{u_{j}^{\prime }}$ is the context vector from node *v*_*i*_. We use only this context vector for calculation. In this study, we are interested in finding the node mapping vector. Then, using this equation, we can formulate the difference from the edge weight on the original network. Letting *P*(⋅|*v*_*i*_) be the distribution of edge weights from node *v*_*i*_ to all nodes (assuming that nodes with no edges are connected with weight 0), then we calculate the distance between the edge weight distribution derived from the vector representation and that on the original network using function *d*. Using this function *d*, we sum over all vertices by the equation $O=\sum_{i=1}^{\left|V\right|}\lambda_{i}d(P\left(\;\cdot \;|v_{i}\right),\hat{P}\left(\;\cdot \;|v_{i}\right))$ and calculate the difference between the distribution on the original network and the distribution from the vector representation obtained. For example, we adopt the KL pseudo-distance for the distance function *d* and the coefficient $\lambda_{i}=\sum_{j=1}^{\left|V\right|}w_{ij}$. We specifically examine the variable part of $\hat{P}\left(\;\cdot \;|v_{i}\right)$. We can approximate *O* as follows.

$$\begin{array}{c} O\approx -\sum_{(i,j)\in E}w_{ij}\;\text{log}\;\hat{P}\left(v_{j}|v_{i}\right) \end{array}$$ (3)

We compute the sum of distances *O*^*l*^ from the vector set $U^{l}≔\{\vec{u_{i}^{l}}\left|1\leq i\leq \right|V^{l}|\}$ which maps the node set *V*^*l*^ in this particular network *G*^*l*^ to the vector space for all networks in ***G***.

$$\begin{array}{c} O=\sum_{l=1}^{\left|G\right|}O^{l} \end{array}$$ (4)

We define *O* as a loss function and minimize in this expression 4. The mapping vector and the context vector are optimized using the stochastic gradient descent method (SGD.) Therefore, we update for the gradient direction($\frac{\partial O}{\partial \vec{u}}$,$\frac{\partial O}{\partial \vec{u^{\prime }}}$) of each vector. Each vector learns according to the algorithm in Table 1. Here, *T* stands for the number of times of learning, *K* signifies the number of times of negative sampling, and *D* denotes the number of dimensions of the vector space to be mapped. Additionally, we set the learning coefficient to $\rho_{t}=\rho_{0}\frac{1-t}{T}$ and set *ρ*_0_ as initial parameters as in the LINE model [8].

**Table 1:** Learning algorithm.

Learning algorithm
1: **Input**: ***G***, *T*, *ρ*_0_, *K*, *D*. 2: **Output**: ***U***. 3: Initialize the mapping vector (***U***) and the context vector (***U***′) in dimension *D*. 4: **for** *t* = 1 **to** *T* 5: $\rho_{t}=\rho_{0}\left(1-\frac{t}{T}\right)$ 6: **for** *l* = 1 **to** |***G***| 7: Sampling an edge($e_{ij}^{l}$) from the network (*G*^*l*^). 8: Read the mapping vector $\vec{u^{t}}$ and the context vector $\vec{u^{\prime t}}$ corresponding to the node $v_{i}^{l}$ and $v_{j}^{l}$ from ***U***, ***U***′. 9: Update the mapping vector as $\vec{u^{t+1}}=\vec{u^{t}}-\frac{\rho_{t}}{w^{l}}\frac{\partial O}{\partial \vec{u}}$ 10: Update the context vector: $\vec{u^{\prime t+1}}=\vec{u^{\prime t}}-\frac{\rho_{t}}{w^{l}}\frac{\partial O}{\partial \vec{u^{\prime }}}$ 11: END

### 2.4 Identify emerging research areas

The purpose of this paper is extraction of a distributed representation that enables the identification of emerging research areas. Therefore, we set up a task to predict whether an index indicating the results of the research will be in the top *x*% in the future. In this way, we verify the usefulness of distributed representations extracted from a heterogeneous network for predicting future research trends. In this task, we need distributed representations at two points which are current vector space to predict future indices and past vector space to learn each feature weight. When extracting two distributed representation separately, it’s known that each vector space is completely different because each vector is randomly initialized [37]. So, we need to align two vector spaces for weights consistency between current and past vector spaces. To align two vector spaces, our method samples representative entities which can be consistent for a long time. For example, top journals which have high journal impact factors remain top class for a long time. Our method assumes that thus entities is consistent for a long time and adopt the distance between an entity and thus consistent entities as features. Specifically, the following logistic regression predicts some index *I*_*ID*,*Y*+*n*_ of a target (*ID*) after *n* years from a particular year(*Y*).

$$\begin{array}{c} {\hat{I}}_{ID,Y+n}=\sigma (wI_{ID,Y}+{\vec{w}}^{T}\cdot \left(U_{s,Y}^{T}\cdot {\vec{u}}_{ID,Y}\right)) \end{array}$$ (5)

In this equation, *w* and $\vec{w}$ is the weight value and the weight vector to be optimized, ***U***_*s*_ represents a matrix of vectors of the sampled group ***s*** that appropriately samples from entities, and ${\vec{u}}_{ID,Y}$ is the vector learned by the heterogeneous network of a specific entity which ID is *ID* in a particular year *Y*. One can predict whether index *I* is in the top *x*% by learning the weight *w* and $\vec{w}$ using the distance between a specific target ID and the sampled group as a feature. Additionally, it is necessary to learn *w* and $\vec{w}$ at a point in the past (*m* years ago) before year *Y*. Therefore, at the time of training, we use index *I*_*Y*−*m*+*n*_ for *Y* − *m* year to learn. Then, we use the optimized *w* and $\vec{w}$ to predict the index *I*_*Y*+*n*_ and evaluate it.

## 3 Materials

This section describes scholarly dataset to use and the experiment included the settings for distributed representation extraction. We also explain the link prediction conditions that evaluate the characteristics of the extracted distributed representations, and the conditions of the prediction task to identify the emerging research area. Prediction involves four tasks: citation count prediction, author *h*-index prediction, journal impact factor prediction, and research institute’s nature index prediction. Here, we will explain the definition and calculation method of each prediction task and the experimental conditions.

### 3.1 Scholarly dataset

We acquired the dataset from Scopus [38]. We entered “(TITLE-ABS-KEY(nano AND carbon) OR TITLE-ABS-KEY(gan) OR TITLE-ABS-KEY(solar AND cell) OR TITLE-ABS-KEY(complex AND networks)) and PUBYEAR AFT 2006” as a search query and use the relevant literature as a dataset. We call this dataset with “Solar Cell” datset. Though this search query contains words other than solar and cell, the reason is we want to find scientific linkages between other technology fields. We present details of the dataset in Table 2. This dataset includes data on academic literature about Nano Carbon, GaN, complex networks, and solar cells, including those reports published during 2006–2016. There were 342,785 papers published during this period, 5,249,635 papers cited as references, 743,140 authors, 63,485 research institutes, 30,764 journals, and 529,979 keywords. These are all distinct numbers without duplication.

**Table 2:** Dataset overview.

Term	2006–2016
Number of Papers	342,785
Number of Citations	5,249,635
Number of Authors	743,140
Number of Institutes	63,485
Number of Journals	30,764
Number of Keywords	529,979

### 3.2 Conditions in distributed representation extraction

We implement the distributed representation extraction method proposed in Section 2.3 for a heterogeneous network created from scholarly datasets. We set the number of dimensions of the distributed representation to be extracted to *D* = 300, the number of negative examples per sampling to *K* = 5, and the total number of learning to *T* = 10^8^. We also set the initial learning rate to *ρ*_0_ = 0.05.

### 3.3 Conditions for link prediction to evaluate extracted distributed representations

As described in this section, we measure the link prediction between nodes quantitatively to evaluate whether the extracted distributed representation can learn the network structure. In other words, our method learns whether a link exists between a pair of nodes that are randomly selected from the network. We test whether it can sufficiently achieve prediction based on the test data. Our method determines the presence or absence of a link using the formula ${\hat{e}}_{ij}=\sigma (w{\vec{u}}_{i}^{T}\cdot {\vec{u}}_{j})$. It is determined that an edge exists between the two nodes if ${\hat{e}}_{ij}\geq P_{th}$. For training, our method samples a total of 40M pairs of nodes with edges as positive examples and nodes without edges as negative ones from the network. Actually, 10% of all data are test data. We make ten test datasets by this procedure. We predict the presence or absence of an edge between a pair of test data using *w* optimized using training data, and evaluate its accuracy. As a method of comparison, we apply a method of randomly selecting the presence or absence of an edge between nodes at 0.5 probability.

### 3.4 Predictive tasks for identifying emerging research areas

**Fig 3:**
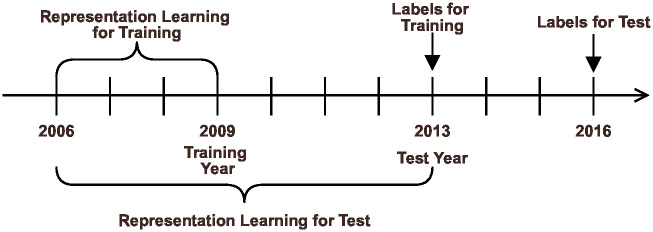
Relation between training period and test period in each index prediction.

In the prediction task for identifying emerging research areas described in Section 2.4, the logistic regression coefficient $\vec{w}$ learned *m* years ago is applied to vector representation ***U***_*s,Y*_ and ${\vec{u}}_{ID,Y}$ as of year *Y*. We predict the index ${\hat{I}}_{ID,Y+n}$ after *n* years. We set *Y* = 2013, *n* = 3, m = 4, and show in Fig 3 the relationship between training duration and training labels versus test duration and test labels. We obtained a heterogeneous network distributed representation extracted using the same settings as those described in Section 3.2. That is, we obtain **U**_2009_ and **U**_2013_, learn $\vec{w}$ using index **I**_2012_ as the correct label, and predict ${\hat{I}}_{2016}$. We assign the index **I** value of each node corresponding to the task to 0, 1. We give 1 for nodes that are in the top 10% (*x* = 0.1) of a particular year’s score, and 0 for nodes that are not. Control group ***s*** is a node group with the top 10% score in the *Y* − *m* year of the task.

#### 3.4.1 Future citation count prediction

The number of citations is the number of times the paper has been cited in other papers. The fact that authors of many papers cite the paper indicates that the ideas in the paper have attracted attention and that the paper is important for the field. The number of citations *Citation*(*Paper*, *N*) after *N* years for a paper *Paper* published in a year *Y* can be calculated as shown below.

$$\begin{array}{c} Citation\left(Paper,N\right)=\sum_{n=0}^{N}\sum_{d}^{D_{Y+n}}Reference\left(d,Paper\right) \end{array}$$ (6)

Actually, *D*_*Y*_ represents the group of papers published that year; d represents each paper. *Reference*(*d*, *Paper*) is a function that returns 1 if paper *d* references paper *Paper*, and 0 otherwise. The number of citations *Citation*(*Paper*, *N*) is the sum of *Reference*(*d*, *Paper*) applied to all papers published within *N* years after the paper was published. By predicting how many times this citation will increase after publication, one can ascertain whether a research paper is fundamentally important immediately after publication. One can identify new studies based on research papers if one can accurately predict future citation counts. Table 3 presents the number of citations in the scholarly dataset published in 2013, in descending order of 2016 citations calculated using this formula. The distribution of citations is very biased. Most papers have few citations. The first paper A in the 2016 ranking was published in 2013, describing production of stable perovskite solar cells and improving energy conversion efficiency by about 15%. In addition, the number of Scopus citations as of 2019 was 6,687. Other papers also show numerous citations on Scopus. The citation ranking of papers on solar cells is almost identical to the actual ranking.

**Table 3:** Ranking of citations for papers published in 2013 in the dataset.

Ranking @2016	Authors	Title	Journal	No. Cited		Ref.
				Dec. 2014	Dec. 2016	
1	J. Burschka, *et al*.	Sequential deposition as a route to high-performance perovskite-sensitized solar cells.	Nature	573	2,320	[39]
2	M. Liu, *et al*.	Efficient planar heterojunction perovskite solar cells by vapour deposition.	Nature	401	1,860	[40]
3	S. D. Stranks, *et al*.	Electron-hole diffusion lengths exceeding 1 micrometer in an organometal trihalide perovskite absorber.	Science	248	1,423	[41]
4	J. You, *et al*.	A polymer tandem solar cell with 10.6% power conversion efficiency.	Nature Communications	619	1,415	[42],
5	G. Xing, *et al*.	Long-range balanced electron-and hole-transport lengths in organic-inorganic CH3NH3PbI3.	Science	219	1,161	[43],
6	J. H. Noh, *et al*.	Chemical management for colorful, efficient, and stable inorganic-organic hybrid nanostructured solar cells.	Nano Letters	212	848	[44],
7	J. H. Heo, *et al*.	Efficient inorganic-organic hybrid heterojunction solar cells containing perovskite compound and polymeric hole conductors.	Nature Photonics	215	768	[42],
8	J. M. Ball, *et al*.	Low-temperature processed meso-superstructured to thin-film perovskite solar cells.	Energy&Environmanetal Science	186	616	[45],
9	H. J. Snaith, *et al*.	Perovskites: The emergence of a new era for low-cost, high-efficiency solar cells.	J. Phys. Chem. Lett.	151	596	[46],
10	P. Docampo, *et al*.	Efficient organometal trihalide perovskite planar-heterojunction solar cells on flexible polymer substrates.	Nature Communications	96	518	[47],

#### 3.4.2 Author *h*-index prediction

The author *h*-index is an index devised by physicist George E. Hirsch based on “Times Cited” of Web of Science [28]. It represents the relative contributions of the respective scientists. As described in this paper, we calculate *h*-index(*h*_*Author*,*Y*_) for a particular author *Author* at year *Y* as the following formula.

$$\begin{array}{c} h_{Author,Y}=\text{min}\left(\text{max}\left(Citations\left(Author,Y,i\right),i\right)\right) \end{array}$$ (7)

Here, *Citations*(*Author*, *Y*, *i*) is a function that returns the number of citations of the paper with the *i*-th largest number of citations by a particular year Y written by the *Author*. In other words, *h*-index *h*_*Author*,*Y*_ means that there are at least *h*_*Author*,*Y*_ papers with more than *h*_*Author*,*Y*_ citations.

Table 4 shows the results obtained from calculating the *h*-index index using this formula in the dataset. Michael-Graätzel, a first-ranked inventor, was the inventor of dye-sensitized solar cells. His work achieved an energy conversion efficiency of 15%, the highest record in dye-sensitized solar cells in 2016. He ranked 253 in *h*-index on Google Scholar in January 2019. Other researchers also showed a large *h*-index. The ranking of *h*-index for solar cell researchers is almost consistent with the actual ranking presented by Google Scholar.

**Table 4:** *h*-index ranking in the dataset.

Ranking @2016	Author	*h*-index		
		2009	2013	2016
1	Michael Graätzel	23	72	116
2	Mohammad K.haja Nazeeruddin	13	45	80
3	Anders Hagfeldt	13	50	74
4	Shaik M.ohammed Zakeeruddin	15	47	64
5	Henry J. Snaith	7	29	63
6	Li Cheng Sun	13	44	60
7	Yong-fang Li	7	34	57
8	Christoph J. Brabec	12	33	57
9	Alan J. Heeger	8	32	55
10	Frederik Christian Krebs	12	41	55

#### 3.4.3 Journal impact factor prediction

The JIF index measures the impact and citation frequency of academic journals in the fields of natural science and social science. We calculate JIF *JIF*(*Journal*, *Y*) for year *Y* in the journal *Journal*, using the following formula.

$$\begin{array}{c} \begin{array}{cc} & JIF(Journal,Y)=\\ & \frac{Citations(Journal,Y-2,Y)+Citations(Journal,Y-1,Y)}{Publications(Journal,Y-2)+Publications(Journal,Y-1)} \end{array} \end{array}$$ (8)

As shown in that formula, *Citations*(*Journal*, *Y* − *n*, *Y*) is the total number of citations at year *Y* for papers published from journal *Journal* in year *Y* − *n*. *Publications*(*Journal*, *Y* − *n*) represents the number of papers published by Journal *Journal* in *Y* − *n* year. In other words, this is the average number of citations at year *Y* for papers published in the past two years from year *Y*.

Table 5 presents the results of calculating JIF using this formula in the dataset. Nature Photonics, number one, is an international journal published by the Nature Publishing Group. It is widely recognized as the top journal among journals specializing in photonics and optoelectronics. The JIF in 2018 is 31.6. Other journals also show large journal impact factor values. The ranking of journal impact factor values in the research field related to solar cells is consistent with the recognition of experts in this research field.

**Table 5:** Journal Impact Factor (JIF) ranking in the dataset.

Ranking @2016	Journal	Imapact Factor		
		2009	2013	2016
1	Nature Photonics	3.4	65.15	98.0
2	Science	8.7	33.6	65.0
3	Nature Chemistry	–	10.5	51.6
4	Nature Materials	19.4	19.6	49.7
5	Chemical Reviews	23.5	3.4	41.8
6	Nature Nanotechnology	1.0	29.1	41.5
7	Physics Reports	–	8.0	30.7
8	Energy and Environmental Science	3.0	11.14	27.3
9	Nature	4.5	8.4	24.1
10	Journal of the American Chemical Society	5.8	14.1	23.1

#### 3.4.4 Nature Index (AC/FC) prediction

The NI is a measure of the influence of research institutes published every year by Nature Publishing Group. It is a general index for measuring the impact of research institutes. Actually, NI has AC, FC, and AC / FC. Then we calculate the following.

$$\begin{array}{ccc} AC(Institute,Y) & = & \sum_{d}^{D(Institute,Y)}\sum_{a}^{Authors(d)}Affiliation\left(a,Institute\right)\\ FC(Institute,Y) & = & \sum_{d}^{D(Institute,Y)}\sum_{a}^{Authors(d)}\frac{Affiliation(a,Institute)}{N(Authors(d))}\\ ACFC(Institute,Y) & = & \frac{AC(Institute,Y)}{FC(Institute,Y)} \end{array}$$

As shown therein, *D*(*Institute*, *Y*) represents the set of papers published by the research institute *Institute* in year *Y*. *Authors*(*d*) denotes the set of authors of a paper *d*. *Affiliation*(*a*, *Institute*) is a function that returns 1 if an author *a* is affiliated with the research institute *Institute*, and 0 otherwise. *N*(*Authors*(*d*)) represents the number of authors of a paper. In other words, AC is an acronym representing the Article(Paper) Count, which assigns 1 point to each institute with which all authors are affiliated. FC is an acronym representing the Fractional Count. Each paper gives the percentage of authors affiliated with a specific institute among all authors. For example, if all authors of a paper are affiliated with the same research institute, then one point is added to that research institute. Actually, ACFC is AC divided by FC. The larger this value becomes, the greater the number of publications are involved, indicating that it is co-authored with authors affiliated with other institutes, indicating greater diversity of the research institute.

Table 6 presents the results of calculating NI AC/FC using this formula in the dataset. The actual NI is calculated only for journals published in the Nature Publishing Group, but we compute NI using all journals included in the dataset. We can calculate NI at various granularities, such as countries, universities, and faculties. Because of the characteristics of the dataset, we calculate NI at the granularity of the university faculty.

**Table 6:** Nature Index AC/FC ranking in the dataset.

Ranking @2016	Institute	Nature Index AC/FC		
		2009	2013	2016
1	Department of Mathematics and Statistics, University of Massachusetts at Amherst	1.09	1.95	4.83
2	Catalan Institution for Research and Advanced Studies (ICREA)	4.27	4.34	4.62
3	Divisions of Human Biology and Public Health Sciences, Howard Hughes Medical Institute,Fred Hutchinson Cancer Research Center	1.70	2.57	4.23
4	National Research University of Information Technologies, Mechanics, and Optics (ITMO), International Laboratory of Metamaterials	–	–	4.14
5	Joint Center for Artificial Photosynthesis and Materials Sciences Division, Lawrence Berkeley National Laboratory	2.75	3.25	4.13
6	Condensed Matter Physics and Materials Sciences Department, Brookhaven National Laboratory	2.04	3.09	4.05
7	Key Laboratory of Biomedical Information Engineering of the Ministry of Education, Frontier Institute of Science and Technology, Xi’an Jiaotong University	1.59	1.76	3.85
8	CAS Key Laboratory of Nanosystem and Hierarchical Fabrication CAS Center for Excellence in Nanoscience National Center for Nanoscience and Technology	1.71	2.37	3.82
9	Department of Chemistry and Nano Science, Ewha Womans University	1.14	2.55	3.82
10	Laboratory of Resources Environment and Geographic Information System, Capital Normal University	–	1.67	3.80

For the multiple tasks described up to this piont, we calculate the index from within the dataset using the equations defined in this section. Furthermore, we evaluate how well the obtained distributed representation can predict these indices.

## 4 Results

This section presents a description of the results. First, we describe the heterogeneous network that has been created. Next, we explain the results of the extracted distributed representation and the consequences of link prediction, which is an evaluation of its characteristics. Finally, we describe the experimentally obtained results of the prediction task to identify new research. Performance of four prediction tasks is assessed: future citation count prediction, author *h*-index prediction, journal impact factor prediction, and Nature index prediction. We explain each result.

### 4.1 Heterogeneous network

The created heterogeneous network has five bipartite networks which are the “Citation Network,”, the “Paper–Journal Network,” the “Paper–Keyword Network,” the “Paper–Author Network,” and “Author–Institute Network.” We show the summary of each bipartite networks in Table 7. In this table, the “Citation Network” has the maximum number of nodes. Also, the “Paper–Journal Network” has the minimum number of nodes. “Paper–Keyword Network” has the highest average node degree. The largest number of nodes in the largest connected component in a network is the “Paper–Paper network.” The smallest one is the “Paper–Journal network.” This demonstrates that the keywords representing the paper content are shared and linked in various articles, but the journal publishes various topics from a single journal.

**Table 7:** Summary of the created heterogeneous network.

Bipartite Network	nodes	edges	largest connected component	average node degree
Citation Network	2,884,616	5,777,364	2,828,458	4.048
Paper–Journal Network	204,264	183,363	3,225	1.999
Paper–Keyword Network	496,034	1,830,474	495,891	7.382
Paper–Author Network	583,225	802,275	318,386	3.527
Author–Institute Network	428,488	475,363	375,400	2.335

### 4.2 Extracted distributed representation

**Fig 4:**
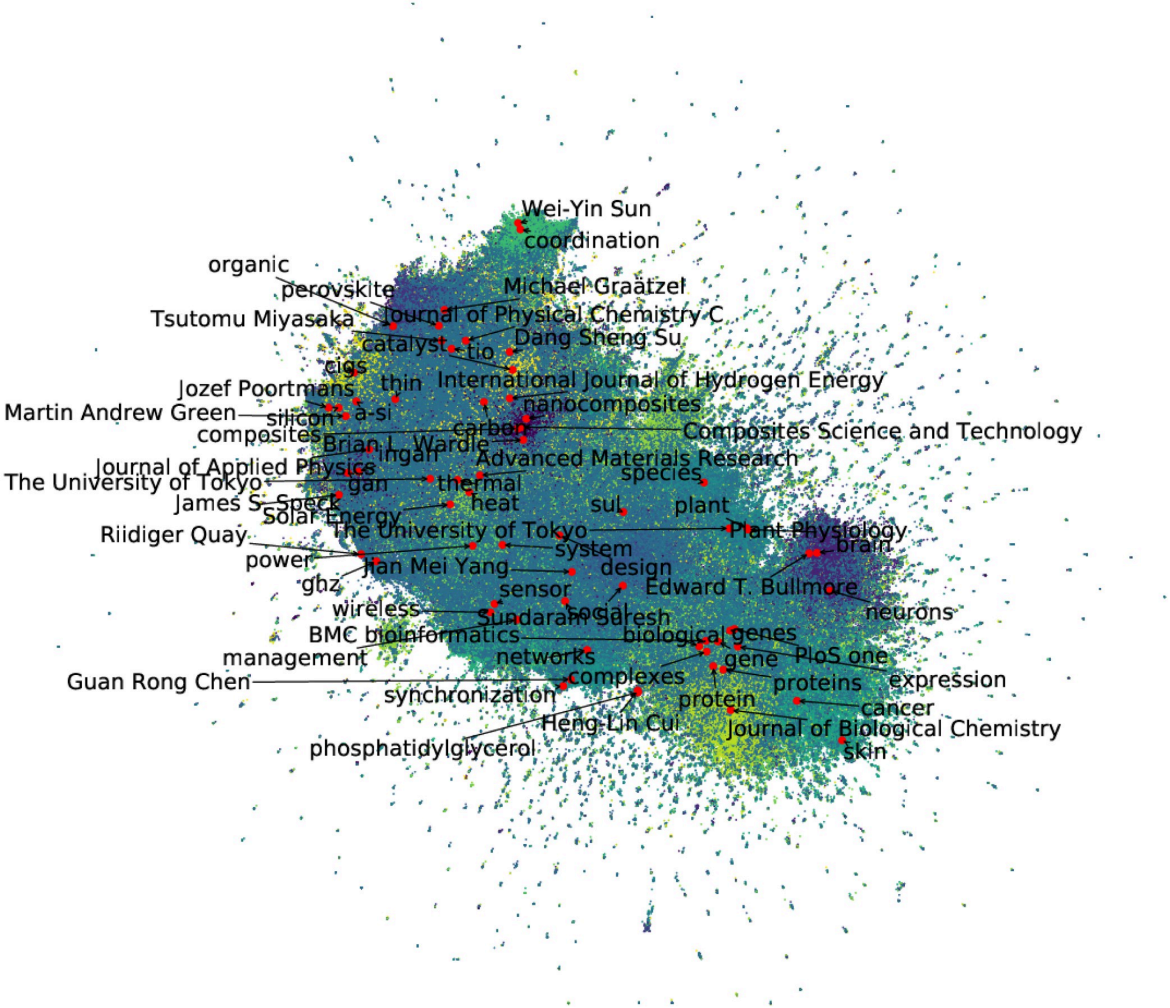
UMAP visualization of acquired distributed representation (color-coded results obtained using K-means method).

Fig 4 presents results of visualization of the extracted distributed representation. The figure shows reduction to two dimensions using the UMAP method [48]. This method makes it possible to reduce the acquired high-dimensional distributed representation to a lower dimension quickly. We can visualize the distributed representation extracted from a large heterogeneous network better using this method. Each point in the diagram represents a node in each heterogeneous network. The text shown in the figure is the label of the entity represented by each node, indicating the names of researchers, research institutes, journals, and research keywords. First, we qualitatively evaluate the validity of this figure. For example, Tsutomu Miyasaka of Toin University of Yokohama invented a perovskite solar cell. Furthermore, he is located near the keyword *perovskite* in the figure. The obtained distributed expression was appropriate as the distribution of research fields and entities related to solar cells.

Next, we quantitatively evaluate the validity of the extracted distributed representation. In Table 8, we show the experimentally obtained results of link prediction between nodes conducted under the conditions described in section 3.3. First, we compare the “Random Network” with others to underscore the effectiveness of the distributed network representation. The “Random Network” has an *F* value of 0.702 ± 0.004; the next smallest value is 0.934 ± 0.004, found for the “Citation Network”. The difference between these two networks has the statistical significance result from the *t*-test. In other words, the obtained distributed representation has the property by which, in the link prediction, smaller distances between nodes are associated with greater numbers of mutual links. Next, to underscore the effectiveness of using the heterogeneous network, we compare “Citation Network” with “Hetero Network.” We compare the *F* values, which reveals 0.934 ± 0.004 for the “Citation Network” and 0.953 ± 0.003 for the “Hetero Network.” The difference between these two networks has the statistical significance result from the *t*-test. These results indicate that distributed expression extraction using the heterogeneous network is more effective in link prediction than distributed expression extraction using a single network.

**Table 8:** Comparison results of link prediction by distributed representation obtained from first-order connection.

Method	Precision	±*σ*	Recall	±*σ*	*F* value	±*σ*
Random Network	0.540	±0.005	1.000	±0.000	0.702	±0.004
Citation Network	0.973*	±0.012	0.898*	±0.007	0.934*	±0.004
Hetero Network	**0.977** *	±0.006	**0.931** * †	±0.006	**0.953** * †	±0.003

* means *t*-test(*P* < 0.01) compared to “Random Network”.

^†^ means *t*-test(*P* < 0.01) compared to “Citation Network”.

### 4.3 Predictive tasks for identifying emerging research areas

#### 4.3.1 Future citation count prediction

We present the AUC of the prediction result in Table 9 and Precision, Recall, and F values in Table 10. First, we explain Table 9. Actually, “Baseline” is a random prediction of whether the number of citations falls in the top 0.1%; it is 0.497 in citation number prediction. By contrast, “Proposed” is 0.739. In prediction of the number of citations, “Proposed” exceeded the baseline by about 0.2 points. Next, we explain the Precision, Recall, and F value results presented in Table 10. First, in “baseline”, the F value is 0.428. Next, at “proposed@*P*_*th*_ = 0.42”, the F value is 0.456. This *P*_*th*_ = 0.42 is the point showing the highest F value.

**Table 9:** Results of emerging research area identification (AUC).

Method	Number of Citation	*h*-index	JIF	NI(ACFC)
Proposed	**0.739**	**0.969**	**0.746**	**0.659**
baseline	0.497	0.852	0.735	0.599

**Table 10:** Results of future citation prediction.

Method	*P*_th_	Precision	Recall	F value
proposed	0.42	0.434	**0.480**	**0.456**
proposed	0.80	**0.766**	0.034	0.066
random	0.50	0.533	0.358	0.428

#### 4.3.2 Author *h*-index prediction

We show the AUC of the prediction result in Table 9 and the actual Precision, Recall, and F values in Table 11. First, we explain Table 9. There, “Baseline” predicts whether *h*-index is in the top 0.1% based on whether it is in 2013 or not. It is 0.852 for *h*-index prediction. Here, “Proposed” is 0.969. The results for *h*-index prediction show that it is about 0.11 points above the “baseline.” Next, we explain the Precision, Recall, and F values shown in Table 11. According to *h*-index in 2013, the F value is 0.748. Next, for “proposed@*P*_*th*_ = 0.50”, the F value is 0.748. This “*P*_*th*_ = 0.50” is the point that shows the highest F value.

**Table 11:** Results of future *h*-index prediction.

Method	*P*_th_	Precision	Recall	F value
Proposed	0.50	0.757	0.739	0.748
*h*-index@2013	0.50	0.757	0.739	0.748

#### 4.3.3 JIF prediction

We show the AUC of the prediction results in Table 9 and the Precision, Recall, and F values in Table 12. First, we explain Table 9. “Baseline” predicts whether the journal impact factor is in the top 0.1% by 2013 or not; it is 0.735 in the JIF prediction. “Proposed” is 0.746. In JIF prediction, it is about 0.011 points above the “baseline.” Next, we explain the actual Precision, Recall, and F values presented in Table 12. At 2013 JIF, the F value is 0.554. Next, at “proposed@*P*_*th*_ = 0.17”, the F value is 0.286. This “*P*_*th*_ = 0.17” is the point showing the highest F value.

**Table 12:** Results of future JIF prediction.

Method	*P*_th_	Precision	Recall	F value
proposed	0.17	0.586	0.189	0.286
proposed	0.80	**0.684**	0.060	0.110
JIF@2013		0.486	**0.645**	**0.554**

#### 4.3.4 Nature Index (AC/FC) prediction

We show the AUC of the prediction results in Table 9, in addition to the Precision, Recall, and F values in Table 13. First, we explain Table 9. Actually, “baseline” predicts whether the Nature Index is in the top 0.1% by 2013 or not; it is 0.599 in the Nature Index prediction. “Proposed” is 0.659. In NI AC/FC prediction, it is about 0.06 points above the baseline. Next, we explain the Precision, Recall, and F values shown in Table 13. First, at 2013 NI, the F value is 0.426. Next, for “proposed@*P*_*th*_ = 0.13”, the F value is 0.461. This “*P*_*th*_ = 0.13” is the point representing the highest F value.

**Table 13:** Results of future NI AC/FC prediction.

Method	*P*_th_	Precision	Recall	F value
proposed	0.13	0.314	**0.865**	**0.461**
proposed	0.80	**0.786**	0.059	0.111
NI AC/FC@2013		0.410	0.443	0.426

## 5 Discussion

Here we discuss the results presented in Section 4. First, we consider the distributed representation obtained using the proposed method applied to the created heterogeneous network. For example, in the Paper–Journal network, the ratio of the maximum number of connected nodes is about 1.5% of the actual number of nodes. In such a sparse network, it is difficult to compare the relation with unconnected nodes using only a single network. However, using the proposed method, it is possible to compare the relation with nodes that are not connected in a sparse network through multiple heterogeneous networks. An illustrative comparison is that of “Journal of Physical Chemistry C” and “Journal of Biological Chemistry” in Fig 4, which can be used to clarify the distributed representations that were obtained. First, it can be confirmed that “Journal of Physical Chemistry C” and “Journal of Biological Chemistry” are located far apart. In addition, “Journal of Physical Chemistry C” has many keywords related to materials such as *perovskite* and *TiO_2_*, whereas “Journal of Biological Chemistry” has biochemistry such as *cancer* and *skin*. These keywords represent the contents that are specific to each journal. Using the proposed method in this way, we can extract relations between nodes that cannot be compared using an extremely sparse Journal–Paper network alone. The comparison results for link prediction in Table 8 confirm the possibility of obtaining a suitable node distributed representation using distributed representation extraction with heterogeneous networks rather than using a single network.

From the results presented above, the heterogeneous network distributed representation extracted by the proposed method appropriately represents the positional relation between nodes.

Next, we discuss prediction tasks for identifying emerging research areas. As a prediction task for identifying an emerging research area, this paper calculates the future citation count of the paper, the author’s future *h*-index prediction, the future journal impact factor prediction, and the future nature index prediction. Four tasks were performed. A great difference exists between the number of cited papers and other tasks. That is, when the paper is published, the paper content is fixed. Still, the information of the author, journal, and research institute is updated variously depending on the year. In this respect, the citation count prediction of papers and other tasks differ. Therefore, we used random prediction as a comparison target for citation count prediction of the paper. However, for other tasks, we selected the value of each index as of 2013. Furthermore, in these three prediction tasks, the value of the target index at the past time is added as a feature value. In Table 9, which summarizes the results of each task, the proposed method achieves better results in all cases.

From the above, it is apparent that the heterogeneous network distributed representation extracted using the proposed method is a feature that functions effectively to identify new research fields. The degree of the effect depends on the task characteristics. In the citation count prediction, the proposed method improvement is 0.24 points, but in other tasks, the AUC is improved from 0.06 points to 0.11 points. This difference indicates the following. Although the proposed method can map the status at a specific point in time (citation prediction task), it does not allow mapping that incorporates changes from the past point in time (*h*-index prediction, journal impact factor prediction, nature index prediction). It will be necessary to embed some consideration of network dynamics to discover an emerging research area more effectively. As described in this paper, we conducted experiments using a dataset related to “solar cell.” This dataset is related to a small number of academic fields of a massive amount of scholarly data. Future studies will be undertaken to broaden the scope of the academic field of interest. Also in Section 2.4, our method predicts a future index prediction by calculating the similarity between sampled nodes’ vector and the target node. This is because the trained vector space is inconsistent in the time direction. We need to develop an end-to-end training method to considering a vector space consistency in time direction and identifying emerging research areas.

In this paper, we predicted four research indicators, which are the citation count, the author *h*-index, the journal impact factor, and the nature index. We select the most popular research indicators related to each research object such as citation, author, journal, and affiliation. However, it is noted that the research indicators we used in this study are abused and they are not the only optimal research indicators [23, 24, 26]. The main argument is that these research indicators does not measure the quality of a given research. On the other hand, there are efforts to resolve the problems that research indicators have [49]. For example, Pudovkin et.al. proposed a normalized journal impact factor that corrects for differences in citation numbers between research fields [25]. Our proposed method is applicable to thus improved research indicators and reduce the risk of reliance on specific indicators.

Our methodology also makes certain critical studies challenging to find. These are studies of endemic diseases in specific regions and studies of sociology in non-English-speaking countries. Because developing medicines in poor regions usually does not get many citations or are published in top cited journals.

## 6 Conclusion

In this paper, we conducted predictive analysis of multiple future scientific impacts by embedding a heterogeneous network. First, we demonstrated that by constructing a heterogeneous network and extracting distributed representations, one could obtain higher accuracy in link prediction between nodes than in a single network in Table 8. Furthermore, we demonstrated that mapping all entities to the same vector space using a node embedding method can predict future research indices in Table 9. Also, we found the top entities in the visualization result in Fig 4. From the above, we conclude that the network embedding method is one of the promising tools for capturing and expressing scientific trends because this method can integrate multiple prediction models for scientific indices.

In future work, we need to develop methods that can predict more diverse aspects of research importance. As mentioned in the discussion section, the proposed method cannot predict the importance of some studies, such as endemic studies, from our heterogeneous network. In order to deal with them correctly, we need to develop a method integrated with natural language processing to consider of the content of the paper, however the specific method is still unknown. We will add social networks to the proposed heterogeneous network and potentially add funding information to evaluate social impacts of a paper. We also have the potential to improve the uniformity aspect of research indicator predictions by removing bias effect or by adding indicators that assess social impact, such as Altmetrics.
